# Professor Elkanah Williams, M. D.

**Published:** 1888-11

**Authors:** 


### OBITUARY.

Professor Elkanah Williams, M. D., of Cincinnati, died at Hazelwood, Pennsylvania, October 5, 1888. He was born in Bedford, Indiana, December 19, 1822. He graduated in medicine in Louisville, Kentucky, and located in Cincinnati. Soon after Professor Helmholtz made known the ophthalmoscope and its revelations, Dr. Williams visited Europe and devoted himself to the study of diseases of the eye, especially in Germany and France, for nearly three years. In 1855 he returned to Cincinnati, and was soon elected Professor of Ophthalmology in a medical college. The honor has been accorded him of being the first scientific ophthalmologist established in the United States. For more than thirty years he was a successful practitioner in his favorite branch, as well as a teacher and writer of recognized ability. His professional attainments and his personal worth inspired confidence in all who came in contact with him, and his social qualities made friends of all who knew him well. He was a man among men. Two years ago, whilst he was engaged as president-elect in the work of organizing the section of ophthalmology in the Washington International Medical Congress, it was discovered that his health, previously impaired, was rapidly failing, and, on consultation with his medical advisers, it was decided that he should abandon for an indefinite time all work and anxiety. After that decision, in writing to a colleague and expressing his regret at this enforced abandonment of his work in connection with the Congress, in which he took great interest, he said: "Much as I would like to serve the Congress, when I know that it is prejudicial to my health, I feel it my duty to resign; health is precious above all considerations."

For more than a year it has been manifest that his most serious affection was organic disease of the brain, and that was the immediate cause of the death by which America lost her Nestor among scientific ophthalmologists, and within a few months of the demise of his friend and confrère, Professor C. R. Agnew, than whom no two men in the United States did more to secure recognition and respect for scientific specialism in the medical profession. All will concede that in the death of these two great and good men the medical profession and our country have sustained a serious loss.

